# Datasets for supplier selection and order allocation with green criteria, all-unit quantity discounts and varying number of suppliers

**DOI:** 10.1016/j.dib.2017.06.018

**Published:** 2017-06-09

**Authors:** Sadeque Hamdan, Ali Cheaitou

**Affiliations:** Sustainable Engineering Asset Management (SEAM) Research Group and Industrial Engineering and Engineering Management Department, University of Sharjah, 27272 Sharjah, United Arab Emirates

**Keywords:** Green supplier selection data, Computation time data, All-unit quantity discounts, Supplier availability, Bi-objective optimization, Multi-criteria decision-making

## Abstract

This data article provides detailed optimization input and output datasets and optimization code for the published research work titled “Dynamic green supplier selection and order allocation with quantity discounts and varying supplier availability” (Hamdan and Cheaitou, 2017, In press) [1]. Researchers may use these datasets as a baseline for future comparison and extensive analysis of the green supplier selection and order allocation problem with all-unit quantity discount and varying number of suppliers. More particularly, the datasets presented in this article allow researchers to generate the exact optimization outputs obtained by the authors of Hamdan and Cheaitou (2017, In press) [1] using the provided optimization code and then to use them for comparison with the outputs of other techniques or methodologies such as heuristic approaches. Moreover, this article includes the randomly generated optimization input data and the related outputs that are used as input data for the statistical analysis presented in Hamdan and Cheaitou (2017 In press) [1] in which two different approaches for ranking potential suppliers are compared. This article also provides the time analysis data used in (Hamdan and Cheaitou (2017, In press) [1] to study the effect of the problem size on the computation time as well as an additional time analysis dataset. The input data for the time study are generated randomly, in which the problem size is changed, and then are used by the optimization problem to obtain the corresponding optimal outputs as well as the corresponding computation time.

**Specifications Table**

**Table:** 

Subject area	*Engineering Management*
More specific subject area	*Operation research and supply chain management*
Type of data	*Tables, Figures, MATLAB data files, MATLAB codes (.m files), MS Excel file (.xlsx)*
How data was acquired	*Generated using Excel and MATLAB*
Data format	*Raw, analyzed*
Experimental factors	*Not applicable*
Experimental features	*Numerical experiments*
Data source location	*Not applicable*
Data accessibility	*Data are within this article*

**Value of the data**

• The datasets include input and output exact optimization data for the multi-period green supplier selection and order allocation problem with variable supplier availability and all-unit quantity discounts. This data can be used by other researchers for comparison with the heuristic solutions obtained by other methods for the same problem.
• The datasets include a computer optimization code that uses the input data in order to generate and analyze the output data. The optimization code available in the time analysis folder can also generate random input data that can be used for time analysis purposes.
• The time analysis datasets can be used by other researchers to benchmark for the purpose of developing and comparing other algorithms, such as heuristics.
• The datasets include input and output data on two supplier evaluation approaches, mainly based on AHP and fuzzy TOPSIS, which can be used by other researchers for comparison with other methods of supplier ranking.

## 1 Data

The datasets of this article provide additional information to [1] and contains four categories of data (datasets). The first dataset contains the optimization input and output data used in the statistical analysis in section 4.1.4 of [1] to compare between two supplier ranking approaches in a context of varying number of suppliers. The two ranking approaches can be described as follows:

Case A ranking all the suppliers one time at the beginning of the first period, which provides preference weights valid for the entire planning horizon.

Case B ranking in each period only the suppliers available in that period, which provides preference weights valid for that period only.

The second dataset contains the quantities ordered from each supplier in the two previously mentioned cases (Case A and Case B) in each period of the planning horizon based on the numerical example described in section 4.1.2 of [1]. The third dataset summarizes the quantities purchased from each supplier in all periods of the planning horizon for the example mentioned in section 4.2 of [1]. The last dataset provides the input data of the instances used in the time study presented in [1], the computation time data, as well as the MATLAB R201a code files. Moreover, it contains the input and output data of the additional time-study of Section 2.4 of this data article.

The datasets of this article are used for the deterministic bi-objective optimization model which is described in [1]. For clarity and completeness purposes, we give here a brief summary of the model, which makes the use of the data even easier. This model described in [1] is an extension to the models presented in [2] and [3] that deal with the problem of green supplier selection and order allocation using fuzzy TOPSIS, AHP and bi-objective linear programming. This model provides a decision-making tool to select the optimal suppliers, the optimal quantities to be purchased and stored, and the optimal amount of shortage in each period. The model suggests a separation between the green aspects and the non-green aspects (traditional criteria) during the evaluation and ranking of suppliers. Originally, the non-linear programming model of [2] was converted into a multi-objective linear programming model as described in [3] with an extensive analysis of the separation approach and the model configuration (i.e. the use of bi-objective and multi-objective configurations). In addition, the extension of the models of [2] and [3] that is presented in [1], considers all-unit quantity discounts and allows the number of available suppliers to vary between the periods of the planning horizon due to capacity limitations, for instance, which was not allowed in the models presented in [2] and [3].

In general, the data of the optimization instances (input data) include all-unit quantity discount data, i.e. ranges and price breaks for each available supplier, customer demand for the periods of the planning horizon, unit inventory holding cost per period, unit penalty shortage cost per period, suppliers’ preference weight for green criteria and traditional criteria, fixed ordering cost for every supplier and the list of the available suppliers in each period.

**Fig. 1:**
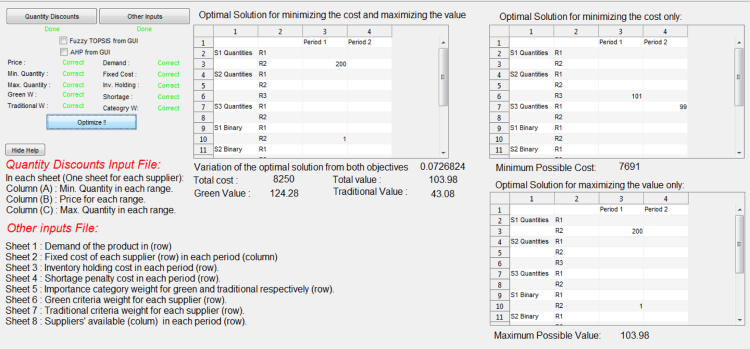
GUI of the software developed using MATLAB R2014a.

The model described in [1] has also been implemented in MATLAB R2014a and the corresponding codes are provided with this data article. To make the developed tool user-friendly, a graphical user interface (GUI) was designed. This GUI requires two MS Excel input files, one includes the quantity discount data (i.e. ranges and prices) and the other one contains the other input data (demand, inventory holding cost, shortage cost, green criteria evaluation, traditional criteria evaluation, etc.). The GUI launches then an optimization code that solves the bi-objective model and the optimization outputs are displayed on a screen, as shown in Fig. 1, and saved into an MS Excel file. Since the GUI was developed using academic MATLAB licenses, it cannot be made available as an open source file. Thus, we provide with this data article only our MATLAB R2014a codes that are used to solve the optimization models.

## 2 Experimental design, materials and methods

### 2.1 Data and code for statistical analysis

The "Statistical Analysis" data folder available in the supplementary materials of this data paper contains two folders: "Case A" and "Case B". Each of these two folders includes the optimization code files, as described in Table 1. They also include all the input and output data, that are stored in subfolders, for the 16 samples that are used to develop Tables 11–14 in [1], as detailed in Table 2.

**Table 1:** Statistical analysis code files.

**File name**	**File type**	**Description**
RunSensetivityAnalysis.m	MATLAB code (.m file)	This MATLAB code performs a sensitivity analysis on one of the model parameters (user defined). This script calls the following scripts: SenAnalysisscript.m, MultiModel5.m, and Evaluate.m
SenAnalysisscript.m	MATLAB code (.m file)	This MATLAB code uses the user defined model parameter and imports its value from the MS Excel file SenAnalysis.xlsx
MultiModel5.m	MATLAB code (.m file)	This MATLAB code calls Data.m to import the data, Model5.m, to solve the optimization model for each objective function separately and then uses the output to solve the combined objectives subject to the defined constraints. This script calls mObjectiveFunction.m
mObjectiveFunction.m	MATLAB function (.m file)	This function script defines the objective functions of the bi-objective model.
Model5.m	MATLAB code (.m file)	This MATLAB code defines all the optimization constraints and calls SingleObjectiveFunction.m to define the objective function and then solves the optimization model for each objective function separately.
SingleObjectiveFunction.m	MATLAB function (.m file)	This function script defines the objective function that will be solved separately.
Data.m	MATLAB code (.m file)	This MATLAB code reads the input data from the MS Excel file (Input.xlsx) while skipping the variable specified for the sensitivity analysis test.
Evaluate.m	MATLAB code (.m file)	This MATLAB code uses the obtained optimal solution to calculate the total cost of purchasing, the total value of purchasing, the total green value of purchasing and the total traditional value of purchasing
EvaluatePercentage.m	MATLAB code (.m file)	This MATLAB code calculates the objective function percentage variation of the bi-objective model.

**Table 2:** Statistical analysis dataset description.

**File name**	**File type**	**Description**
Input.xlsx	MS Excel	An input data file containing 8 sheets. Sheet 1 (D) contains demand data in each period. Sheets 2 and 3 (H and S) contain inventory holding cost and shortage penalty cost in each period respectively. Sheet 4 (CF) is the fixed cost for each supplier (row-wise) in each period (column-wise). Sheet 5 (WAHP) includes the category weights where the first value represents the AHP weight for green category and the second value is for the traditional category – it is worth noting that this sheet is ignored during the statistical analysis and is replaced by SenAnalysis.xlsx. Sheets 6 and 7 (GW and TW) contain the green criteria and traditional criteria preference weights for each supplier (row-wise) respectively and for each period (column-wise) in Case B only. Sheet 8 (List) contains the supplier availability list (row-wise) in each period (column-wise).
QDiscount.xlsx	MS Excel	An input data file that contains all-unit quantity discount information, where each sheet represents a supplier (sheet 1 for supplier 1, sheet 2 for supplier 2 and so-on). In each sheet, the first column represents the minimum ordering quantity in each range, the second column includes the price for the corresponding range and the last column is for the maximum ordering quantity in each range. The different ranges are shown in different rows.
SenAnalysis.xlsx	MS Excel	An input data file that includes the data required for sensitivity analysis calculations, where each case contains the input data (WAHP) for each scenario.
SenAnaEvaluate.xlsx	MS Excel	An output data file containing the evaluation of the optimal solution obtained in each scenario (each sheet). The first value represents the total cost of the solution; the second value represents the total combined value of purchasing (green and traditional); the third and fourth values are the total green value and the total traditional value of purchasing respectively; the last two values represent the total optimal cost and total optimal value of the single objective models respectively.
SenAnaFval.xlsx	MS Excel	An output data file that stores the optimal variation of the solution in each scenario (each sheet)
SenAnaResults.xlsx	MS Excel	An output data file that stores the optimal ordering schedule in each scenario (each sheet). In each sheet, the column indicates the period, the first row indicates the quantities purchased from the first price range of supplier 1, the second row contains the quantities ordered from the second range of supplier 1 and so forth, then the quantities ordered from the first range of supplier 2, etc… The following similar number of rows contain the binary variables for each price range of each supplier. The last four rows represent the inventory levels at the end of each period, the shortage quantities of each period, and their corresponding binary variables.

### 2.2 Data for supplier ranking

The "Ranking Approach Comparison" data folder available in the supplementary materials of this data paper contains the data related to the detailed output of the numerical experiments discussed in section 4.1.2 of [1]. The MS Word file (Summary.docx) contains Tables A.1–A.20, one table for each period, that represent a comparison between the quantities purchased from each supplier in Case A and Case B under different scenarios of the importance weights of the two sets of criteria (green and traditional). To avoid duplication, the optimization input data are available in [1].

### 2.3 Data for "quantity discount" vs. "no quantity discount"

The "QD vs No QD" data folder available in the supplementary materials of this data paper includes an MS Word file (QD vs no QD Output.docx) that provides the detailed output data of the numerical experiments presented in section 4.2 of [1]. This numerical experiment investigates the competition between suppliers offering a single variable cost against suppliers with similar characteristics but offering all-unit quantity discounts. Tables B.1–B.5 in the MS Word file mentioned previously, contains the total quantities ordered from each supplier in all periods under different scenarios of the importance weight of the two sets of criteria (green and traditional). The detailed optimization input data of this numerical experiment are available in [1].

### 2.4 Time analysis

**Fig. 2:**
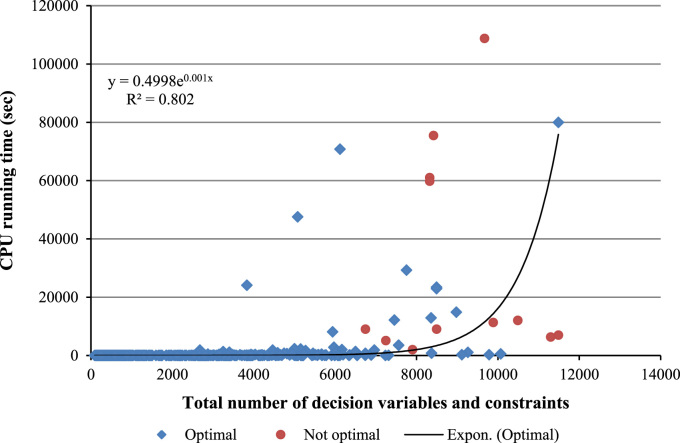
Total number of decision variables and constraints versus CPU running time.

To better estimate and fit the computation time required to solve the optimization model described previously, 560 instances with different sizes in terms of the input parameters have been randomly generated. These instances have then been solved using MATLAB R2014a and their CPU running time has been recorded. The computer used for this data paper is equipped with an Intel(R) Core(TM) i5-4590, CPU @ 3.3 GHz, 8.00 GB RAM, and Microsoft Windows 7 64-bit operating system, which is different from the computer used for the time analysis presented in [1]. The "Time Analysis" data folder available in the Supplementary materials of this data paper includes all the input data of the 560 instances used in the time analysis as MATLAB data files (.mat). The obtained optimization output of all instances is included in the same folder as MS Excel files (.xlsx). Each MS Excel file consists of six sheets whose contents are described in Table 3. Moreover, Table 4 provides a description of the code files used in this dataset as well as in the time analysis presented in Section 4.3 of [1]. Moreover, the MS Excel file (Final_Results.xlsx) included in the same folder summarizes the computation time and instance sizes of the 560 instances. Furthermore, Table 5 provides a sample of the summarized data for 10 instances provided as an illustration. Fig. 2 shows the computation time of the optimization model against the total number of decision variables and constraints for all 560 instances. It is worth noting that the fitting regression line does not take into account the non-optimal points. These points correspond to instances for which the optimal solution could not be found even after the computation time reported in Fig. 2. Finally, Table 6 provides all data used to produce Fig. 9 provided in the time analysis (Section 4.3) in [1].

**Table 3:** Content of the MS Excel files in the time analysis dataset.

**Sheet number**	**Content**
Sheet 1	The optimal solution of the cost single objective problem.
Sheet 2	The optimal solution of value of purchasing single objective problem.
Sheet 3	The optimal solution of the bi-objective problem.
Sheet 4	This sheet contains three values: 1. The optimal total cost corresponding to the solution of sheet 1. 2. The optimal total value of purchasing corresponding to the solution of sheet 2. 3. The variation from the first two values which corresponds to the solution of sheet 3.
Sheet 5	The optimality status of each solution in the first three sheets respectively, where 0 indicates that no solution is found, 1 indicates that the solution is optimal and 2 indicates that the solution is feasible but not optimal.
Sheet 6	This sheet contains four values: 1. The CPU running time. 2. The elapsed time using tic toc function of MATLAB. 3. The number of decision variables. 4. The number of constraints.

**Table 4:** Description of the code files in the time analysis dataset.

**File name**	**File type**	**Description**
Data.m	MATLAB code (.m file)	This MATLAB code generates random instances.
DynamicRun.m	MATLAB code (.m file)	This MATLAB code calls Data.m code to generate instance and then calls MultiModel5 to optimize the generated instance.
MultiModel5.m mObjectiveFunction.m Model5.m SingleObjectiveFunction.m	MATLAB code/ MATLAB function (.m file)	These MATLAB files are described in Table 1.
Summarize_All	MATLAB code (.m file)	This MATLAB code reads all the generated output files (MS Excel files) and classifies them into optimal and non-optimal, then saves the results in another MS Excel file (Final_Results.xlsx).

**Table 5:** Illustrative sample of the time analysis data.

**Number of decision variables**	**Number of constraints**	**Total (number of decision variables + constraints)**	**CPU running time (s)**
2048	2284	4332	46.35
2108	2342	4450	145.77
1992	2215	4207	165.60
2614	2899	5513	265.95
2050	2284	4334	21.26
1972	2186	4158	14.20
2814	3126	5940	8087.06
4274	4705	8979	14839.95
3974	4398	8372	797.66
3296	3669	6965	1863.73

**Table 6:** Summary of the data of the time analysis presented in [1].

**#**	**No. of decision** **variables**	**No. of** **constraints**	**Total**	**Running** **time (s)**	**#**	**No. of decision** **variables**	**No. of** **constraints**	**Total**	**Running** **time (s)**
1	22	55	77	1.435209	46	1156	2498	3654	44.02348
2	22	55	77	1.638011	47	1202	2530	3732	41.04386
3	22	55	77	3.16682	48	1214	2558	3772	26.64497
4	28	68	96	1.372809	49	1216	2700	3916	93.9126
5	30	73	103	1.404009	50	1224	2639	3863	28.86019
6	30	73	103	1.435209	51	1224	2639	3863	76.67449
7	32	77	109	1.154407	52	1278	2751	4029	12.10568
8	40	97	137	1.435209	53	1302	2801	4103	51.07473
9	46	110	156	1.435209	54	1312	2913	4225	151.7734
10	46	110	156	1.48201	55	1370	2942	4312	107.0479
11	48	115	163	1.435209	56	1406	2997	4403	131.5088
12	74	179	253	3.16682	57	1408	3126	4534	208.4953
13	86	206	292	3.385222	58	1408	3126	4534	214.0022
14	98	232	330	3.728424	59	1408	3126	4534	229.6335
15	120	287	407	3.354022	60	1504	3339	4843	31.0754
16	134	318	452	3.822025	61	1504	3339	4843	133.7865
17	148	349	497	3.354022	62	1504	3339	4843	135.3309
18	176	418	594	3.463222	63	1504	3339	4843	292.2679
19	186	440	626	3.666023	64	1530	3274	4804	538.4843
20	212	497	709	2.886018	65	1530	3274	4804	704.4225
21	230	526	756	3.993626	66	1536	3218	4754	51.87033
22	280	633	913	3.385222	67	1536	3410	4946	281.3478
23	410	881	1291	3.962425	68	1536	3410	4946	407.1002
24	518	1103	1621	7.675249	69	1536	3410	4946	442.6528
25	604	1345	1949	18.12732	70	1536	3410	4946	1848.378
26	604	1345	1949	18.82932	71	1584	3386	4970	297.3067
27	604	1345	1949	19.03212	72	1602	3401	5003	44.19508
28	634	1409	2043	24.13335	73	1602	3401	5003	316.8068
29	634	1409	2043	24.67936	74	1602	3401	5003	2816.754
30	640	1422	2062	29.35939	75	1628	3480	5108	1257.165
31	640	1422	2062	30.06139	76	1632	3623	5255	2504.736
32	680	1524	2204	12.07448	77	1632	3623	5255	7111.243
33	680	1524	2204	14.75769	78	1632	3623	5255	7197.418
34	712	1584	2296	42.71307	79	1634	3399	5033	2849.093
35	736	1635	2371	72.52486	80	1696	3765	5461	6223.363
36	928	2061	2989	26.31737	81	1696	3765	5461	7208.525
37	928	2061	2989	63.49241	82	1728	3836	5564	2493.972
38	992	2203	3195	14.21169	83	1728	3836	5564	7046.643
39	992	2203	3195	58.14157	84	1736	3607	5343	7214.609
40	992	2203	3195	75.17688	85	1750	3685	5435	2182.314
41	1056	2345	3401	74.16288	86	2064	4274	6338	1334.62
42	1056	2345	3401	172.9895	87	2064	4274	6338	4972.641
43	1120	2487	3607	57.00277	88	2064	4274	6338	5407.728
44	1120	2487	3607	242.0044	89	2114	4430	6544	2941.024
45	1120	2487	3607	290.0371	90	2448	5114	7562	9640.493

## Appendix A Supplementary material

Supplementary material Statistical analysis data

.

Supplementary material Ranking approach data (Table A.1 - Table A.20)

.

Supplementary material Quantitiy discount vs. No quantity discount data (Table B.1 - Table B.5)

.

Supplementary material Time analysis data
