# Supplementary material for: Datasets for supplier selection and order allocation with green criteria, all-unit quantity discounts and varying number of suppliers
Source: Data Brief. 2017 Jun 9;13:444–52. doi: 10.1016/j.dib.2017.06.018 (PMC5485863; doi:10.1016/j.dib.2017.06.018)
Supplement: Supplementary file 3 — Supplementary material Ranking approach data (Table A.1 - Table A.20) [file mmc3.zip › Data_In_Brief/2.Ranking Approach Comparison/Summary.docx]

**Table A.1. Quantity purchased from each supplier in period 1 in case A and case B**

| Scenario | Case A | | | | Case B | | | |
| --- | --- | --- | --- | --- | --- | --- | --- | --- |
|  | S1 | S2 | S3 | S4 | S1 | S2 | S3 | S4 |
| 0.05 G 0.95 T | 660 | 0 | 0 | 0 | 660 | 0 | 0 | 0 |
| 0.1 G 0.9 T | 660 | 0 | 0 | 0 | 660 | 0 | 0 | 0 |
| 0.15 G 0.85 T | 660 | 0 | 0 | 0 | 660 | 0 | 0 | 0 |
| 0.2 G 0.8 T | 660 | 0 | 0 | 0 | 660 | 0 | 0 | 0 |
| 0.25 G 0.75 T | 660 | 0 | 0 | 0 | 660 | 0 | 0 | 0 |
| 0.3 G 0.7 T | 660 | 0 | 0 | 0 | 660 | 0 | 0 | 0 |
| 0.35 G 0.65 T | 660 | 0 | 0 | 0 | 660 | 0 | 0 | 0 |
| 0.40 G 0.60 T | 660 | 0 | 0 | 0 | 660 | 0 | 0 | 0 |
| 0.45 G 0.55 T | 660 | 0 | 0 | 0 | 660 | 0 | 0 | 0 |
| 0.50 G 0.5 T | 660 | 0 | 0 | 0 | 660 | 0 | 0 | 0 |
| 0.55 G 0.45 T | 660 | 0 | 0 | 0 | 660 | 0 | 0 | 0 |
| 0.60 G 0.40 T | 660 | 0 | 0 | 0 | 660 | 0 | 0 | 0 |
| 0.65 G 0.35 T | 660 | 0 | 0 | 0 | 660 | 0 | 0 | 0 |
| 0.70 G 0.30 T | 660 | 0 | 0 | 0 | 660 | 0 | 0 | 0 |
| 0.75 G 0.25 T | 660 | 0 | 0 | 0 | 660 | 0 | 0 | 0 |
| 0.80 G 0.20 T | 660 | 0 | 0 | 0 | 660 | 0 | 0 | 0 |
| 0.85 G 0.15 T | 660 | 0 | 0 | 0 | 660 | 0 | 0 | 0 |
| 0.90 G 0.10 T | 660 | 0 | 0 | 0 | 660 | 0 | 0 | 0 |
| 0.95 G 0.05 T | 660 | 0 | 0 | 0 | 660 | 0 | 0 | 0 |

**Table A.2. Quantity purchased from each supplier in period 2 in case A and case B**

| Scenario | Case A | | | | Case B | | | |
| --- | --- | --- | --- | --- | --- | --- | --- | --- |
|  | S1 | S2 | S3 | S4 | S1 | S2 | S3 | S4 |
| 0.05 G 0.95 T | 0 | 0 | 0 | 720 | 0 | 0 | 0 | 720 |
| 0.1 G 0.9 T | 0 | 0 | 0 | 720 | 0 | 0 | 0 | 720 |
| 0.15 G 0.85 T | 0 | 0 | 0 | 720 | 0 | 0 | 0 | 720 |
| 0.2 G 0.8 T | 0 | 0 | 0 | 720 | 0 | 0 | 0 | 720 |
| 0.25 G 0.75 T | 0 | 0 | 0 | 720 | 0 | 0 | 0 | 720 |
| 0.3 G 0.7 T | 0 | 0 | 0 | 720 | 0 | 0 | 0 | 720 |
| 0.35 G 0.65 T | 0 | 0 | 0 | 720 | 0 | 0 | 0 | 720 |
| 0.40 G 0.60 T | 0 | 0 | 0 | 720 | 0 | 0 | 0 | 720 |
| 0.45 G 0.55 T | 0 | 0 | 0 | 720 | 0 | 0 | 0 | 720 |
| 0.50 G 0.5 T | 0 | 0 | 0 | 720 | 0 | 0 | 0 | 720 |
| 0.55 G 0.45 T | 0 | 0 | 0 | 720 | 0 | 0 | 0 | 720 |
| 0.60 G 0.40 T | 0 | 0 | 0 | 720 | 0 | 0 | 0 | 720 |
| 0.65 G 0.35 T | 0 | 0 | **0** | **720** | 0 | 0 | **720** | **0** |
| 0.70 G 0.30 T | 0 | 0 | 720 | 0 | 0 | 0 | 720 | 0 |
| 0.75 G 0.25 T | 0 | 0 | 720 | 0 | 0 | 0 | 720 | 0 |
| 0.80 G 0.20 T | 0 | 0 | 720 | 0 | 0 | 0 | 720 | 0 |
| 0.85 G 0.15 T | 0 | 0 | 720 | 0 | 0 | 0 | 720 | 0 |
| 0.90 G 0.10 T | 0 | 0 | 720 | 0 | 0 | 0 | 720 | 0 |
| 0.95 G 0.05 T | 0 | 0 | 720 | 0 | 0 | 0 | 720 | 0 |

**Table A.3. Quantity purchased from each supplier in period 3 in case A and case B**

| Scenario | Case A | | | | Case B | | | |
| --- | --- | --- | --- | --- | --- | --- | --- | --- |
|  | S1 | S2 | S3 | S4 | S1 | S2 | S3 | S4 |
| 0.05 G 0.95 T | 0 | 0 | 0 | 510 | 0 | 0 | 0 | 510 |
| 0.1 G 0.9 T | 0 | 0 | 0 | 510 | 0 | 0 | 0 | 510 |
| 0.15 G 0.85 T | 0 | 0 | 0 | 510 | 0 | 0 | 0 | 510 |
| 0.2 G 0.8 T | 0 | 0 | 0 | 510 | 0 | 0 | 0 | 510 |
| 0.25 G 0.75 T | 0 | 0 | 0 | 510 | 0 | 0 | 0 | 510 |
| 0.3 G 0.7 T | 0 | 0 | 0 | 510 | 0 | 0 | 0 | 510 |
| 0.35 G 0.65 T | 0 | 0 | 0 | 510 | 0 | 0 | 0 | 510 |
| 0.40 G 0.60 T | 0 | 0 | 0 | 510 | 0 | 0 | 0 | 510 |
| 0.45 G 0.55 T | 0 | 0 | 0 | 510 | 0 | 0 | 0 | 510 |
| 0.50 G 0.5 T | 0 | 0 | 0 | 510 | 0 | 0 | 0 | 510 |
| 0.55 G 0.45 T | 0 | 0 | 0 | 510 | 0 | 0 | 0 | 510 |
| 0.60 G 0.40 T | 0 | 0 | 0 | 510 | 0 | 0 | 0 | 510 |
| 0.65 G 0.35 T | 0 | 0 | 0 | 510 | 0 | 0 | 0 | 510 |
| 0.70 G 0.30 T | 0 | 0 | 0 | 510 | 0 | 0 | 0 | 510 |
| 0.75 G 0.25 T | 0 | 0 | 0 | 510 | 0 | 0 | 0 | 510 |
| 0.80 G 0.20 T | 0 | 0 | 0 | 510 | 0 | 0 | 0 | 510 |
| 0.85 G 0.15 T | 510 | 0 | 0 | 0 | 510 | 0 | 0 | 0 |
| 0.90 G 0.10 T | 510 | 0 | 0 | 0 | 510 | 0 | 0 | 0 |
| 0.95 G 0.05 T | 510 | 0 | 0 | 0 | 510 | 0 | 0 | 0 |

**Table A.4. Quantity purchased from each supplier in period 4 in case A and case B**

| Scenario | Case A | | | | Case B | | | |
| --- | --- | --- | --- | --- | --- | --- | --- | --- |
|  | S1 | S2 | S3 | S4 | S1 | S2 | S3 | S4 |
| 0.05 G 0.95 T | 0 | 0 | 0 | 810 | 0 | 0 | 0 | 810 |
| 0.1 G 0.9 T | 0 | 0 | 0 | 810 | 0 | 0 | 0 | 810 |
| 0.15 G 0.85 T | 0 | 0 | 0 | 810 | 0 | 0 | 0 | 810 |
| 0.2 G 0.8 T | 0 | 0 | 0 | 810 | 0 | 0 | 0 | 810 |
| 0.25 G 0.75 T | 0 | 0 | 0 | 810 | 0 | 0 | 0 | 810 |
| 0.3 G 0.7 T | 0 | 0 | 0 | 810 | 0 | 0 | 0 | 810 |
| 0.35 G 0.65 T | 0 | 0 | 0 | 810 | 0 | 0 | 0 | 810 |
| 0.40 G 0.60 T | 0 | 0 | 0 | 810 | 0 | 0 | 0 | 810 |
| 0.45 G 0.55 T | 0 | 0 | 0 | 810 | 0 | 0 | 0 | 810 |
| 0.50 G 0.5 T | 0 | 0 | 0 | 810 | 0 | 0 | 0 | 810 |
| 0.55 G 0.45 T | 0 | 0 | 0 | 810 | 0 | 0 | 0 | 810 |
| 0.60 G 0.40 T | 0 | 0 | 0 | 810 | 0 | 0 | 0 | 810 |
| 0.65 G 0.35 T | 0 | 0 | 0 | 810 | 0 | 0 | 0 | 810 |
| 0.70 G 0.30 T | 0 | 0 | 0 | 810 | 0 | 0 | 0 | 810 |
| 0.75 G 0.25 T | 0 | 0 | 0 | 810 | 0 | 0 | 0 | 810 |
| 0.80 G 0.20 T | 0 | 0 | 0 | 810 | 0 | 0 | 0 | 810 |
| 0.85 G 0.15 T | **810** | 0 | 0 | 0 | 0 | 0 | 0 | **810** |
| 0.90 G 0.10 T | 810 | 0 | 0 | 0 | 810 | 0 | 0 | 0 |
| 0.95 G 0.05 T | 810 | 0 | 0 | 0 | 810 | 0 | 0 | 0 |

**Table A.5. Quantity purchased from each supplier in period 5 in case A and case B**

| Scenario | Case A | | | | Case B | | | |
| --- | --- | --- | --- | --- | --- | --- | --- | --- |
|  | S1 | S2 | S3 | S4 | S1 | S2 | S3 | S4 |
| 0.05 G 0.95 T | 0 | 0 | 725 | 0 | 0 | 0 | 725 | 0 |
| 0.1 G 0.9 T | 0 | 0 | 725 | 0 | 0 | 0 | 725 | 0 |
| 0.15 G 0.85 T | 0 | 0 | 725 | 0 | 0 | 0 | 725 | 0 |
| 0.2 G 0.8 T | 0 | 0 | 725 | 0 | 0 | 0 | 725 | 0 |
| 0.25 G 0.75 T | 0 | 0 | 725 | 0 | 0 | 0 | 725 | 0 |
| 0.3 G 0.7 T | 0 | 0 | 725 | 0 | 0 | 0 | 725 | 0 |
| 0.35 G 0.65 T | 0 | 0 | 725 | 0 | 0 | 0 | 725 | 0 |
| 0.40 G 0.60 T | 0 | 0 | 725 | 0 | 0 | 0 | 725 | 0 |
| 0.45 G 0.55 T | 0 | 0 | 725 | 0 | 0 | 0 | 725 | 0 |
| 0.50 G 0.5 T | 0 | 0 | 725 | 0 | 0 | 0 | 725 | 0 |
| 0.55 G 0.45 T | 0 | 0 | 725 | 0 | 0 | 0 | 725 | 0 |
| 0.60 G 0.40 T | 0 | 0 | 725 | 0 | 0 | 0 | 725 | 0 |
| 0.65 G 0.35 T | 0 | 0 | 725 | 0 | 0 | 0 | 725 | 0 |
| 0.70 G 0.30 T | 0 | 0 | 725 | 0 | 0 | 0 | 725 | 0 |
| 0.75 G 0.25 T | 0 | 0 | 725 | 0 | 0 | 0 | 725 | 0 |
| 0.80 G 0.20 T | 0 | 0 | 725 | 0 | 0 | 0 | 725 | 0 |
| 0.85 G 0.15 T | 0 | 0 | 725 | 0 | 0 | 0 | 725 | 0 |
| 0.90 G 0.10 T | 0 | 0 | 725 | 0 | 0 | 0 | 725 | 0 |
| 0.95 G 0.05 T | 0 | 0 | 725 | 0 | 0 | 0 | 725 | 0 |

**Table A.6. Quantity purchased from each supplier in period 6 in case A and case B**

| Scenario | Case A | | | | Case B | | | |
| --- | --- | --- | --- | --- | --- | --- | --- | --- |
|  | S1 | S2 | S3 | S4 | S1 | S2 | S3 | S4 |
| 0.05 G 0.95 T | 0 | 0 | 465 | 0 | 0 | 0 | 465 | 0 |
| 0.1 G 0.9 T | 0 | 0 | 465 | 0 | 0 | 0 | 465 | 0 |
| 0.15 G 0.85 T | 0 | 0 | 465 | 0 | 0 | 0 | 465 | 0 |
| 0.2 G 0.8 T | 0 | 0 | 465 | 0 | 0 | 0 | 465 | 0 |
| 0.25 G 0.75 T | 0 | 0 | 465 | 0 | 0 | 0 | 465 | 0 |
| 0.3 G 0.7 T | 0 | 0 | 465 | 0 | 0 | 0 | 465 | 0 |
| 0.35 G 0.65 T | 0 | 0 | 465 | 0 | 0 | 0 | 465 | 0 |
| 0.40 G 0.60 T | 0 | 0 | 465 | 0 | 0 | 0 | 465 | 0 |
| 0.45 G 0.55 T | 0 | 0 | 465 | 0 | 0 | 0 | 465 | 0 |
| 0.50 G 0.5 T | 0 | 0 | 465 | 0 | 0 | 0 | 465 | 0 |
| 0.55 G 0.45 T | 0 | 0 | 465 | 0 | 0 | 0 | 465 | 0 |
| 0.60 G 0.40 T | 0 | 0 | 465 | 0 | 0 | 0 | 465 | 0 |
| 0.65 G 0.35 T | 0 | 0 | 465 | 0 | 0 | 0 | 465 | 0 |
| 0.70 G 0.30 T | 0 | 0 | 465 | 0 | 0 | 0 | 465 | 0 |
| 0.75 G 0.25 T | 0 | 0 | 465 | 0 | 0 | 0 | 465 | 0 |
| 0.80 G 0.20 T | 0 | 0 | 465 | 0 | 0 | 0 | 465 | 0 |
| 0.85 G 0.15 T | 0 | 0 | 465 | 0 | 0 | 0 | 465 | 0 |
| 0.90 G 0.10 T | 0 | 0 | 465 | 0 | 0 | 0 | 465 | 0 |
| 0.95 G 0.05 T | 0 | 0 | 465 | 0 | 0 | 0 | 465 | 0 |

**Table A.7. Quantity purchased from each supplier in period 7 in case A and case B**

| Scenario | Case A | | | | Case B | | | |
| --- | --- | --- | --- | --- | --- | --- | --- | --- |
|  | S1 | S2 | S3 | S4 | S1 | S2 | S3 | S4 |
| 0.05 G 0.95 T | 0 | 0 | 0 | 1510 | 0 | 0 | 0 | 1510 |
| 0.1 G 0.9 T | 0 | 0 | 0 | 1510 | 0 | 0 | 0 | 1510 |
| 0.15 G 0.85 T | 0 | 0 | 0 | 1510 | 0 | 0 | 0 | 1510 |
| 0.2 G 0.8 T | 0 | 0 | 0 | 1510 | 0 | 0 | 0 | 1510 |
| 0.25 G 0.75 T | 0 | 0 | 0 | 1510 | 0 | 0 | 0 | 1510 |
| 0.3 G 0.7 T | 0 | 0 | 0 | 1510 | 0 | 0 | 0 | 1510 |
| 0.35 G 0.65 T | 0 | 0 | 0 | 1510 | 0 | 0 | 0 | 1510 |
| 0.40 G 0.60 T | 0 | 0 | 0 | 1510 | 0 | 0 | 0 | 1510 |
| 0.45 G 0.55 T | 0 | 0 | 0 | 1510 | 0 | 0 | 0 | 1510 |
| 0.50 G 0.5 T | 0 | 0 | 0 | 1510 | 0 | 0 | 0 | 1510 |
| 0.55 G 0.45 T | 0 | 0 | 0 | 1510 | 0 | 0 | 0 | 1510 |
| 0.60 G 0.40 T | 0 | 0 | 0 | 1510 | 0 | 0 | 0 | 1510 |
| 0.65 G 0.35 T | 0 | 0 | 0 | 1510 | 0 | 0 | 0 | 1510 |
| 0.70 G 0.30 T | 0 | 0 | 1510 | 0 | 0 | 0 | 1510 | 0 |
| 0.75 G 0.25 T | 0 | 0 | 1510 | 0 | 0 | 0 | 1510 | 0 |
| 0.80 G 0.20 T | 0 | 0 | 1510 | 0 | 0 | 0 | 1510 | 0 |
| 0.85 G 0.15 T | 0 | 0 | 1510 | 0 | 0 | 0 | 1510 | 0 |
| 0.90 G 0.10 T | 0 | 0 | 1510 | 0 | 0 | 0 | 1510 | 0 |
| 0.95 G 0.05 T | 0 | 0 | 1510 | 0 | 0 | 0 | 1510 | 0 |

**Table A.8. Quantity purchased from each supplier in period 8 in case A and case B**

| Scenario | Case A | | | | Case B | | | |
| --- | --- | --- | --- | --- | --- | --- | --- | --- |
|  | S1 | S2 | S3 | S4 | S1 | S2 | S3 | S4 |
| 0.05 G 0.95 T | 0 | 0 | 2410 | 0 | 0 | 0 | 2410 | 0 |
| 0.1 G 0.9 T | 0 | 0 | 2410 | 0 | 0 | 0 | 2410 | 0 |
| 0.15 G 0.85 T | 0 | 0 | 2410 | 0 | 0 | 0 | 2410 | 0 |
| 0.2 G 0.8 T | 0 | 0 | 2410 | 0 | 0 | 0 | 2410 | 0 |
| 0.25 G 0.75 T | 0 | 0 | 2410 | 0 | 0 | 0 | 2410 | 0 |
| 0.3 G 0.7 T | 0 | 0 | 2410 | 0 | 0 | 0 | 2410 | 0 |
| 0.35 G 0.65 T | 0 | 0 | 2410 | 0 | 0 | 0 | 2410 | 0 |
| 0.40 G 0.60 T | 0 | 0 | 2410 | 0 | 0 | 0 | 2410 | 0 |
| 0.45 G 0.55 T | 0 | 0 | 2410 | 0 | 0 | 0 | 2410 | 0 |
| 0.50 G 0.5 T | 0 | 0 | 2410 | 0 | 0 | 0 | 2410 | 0 |
| 0.55 G 0.45 T | 0 | 0 | 2410 | 0 | 0 | 0 | 2410 | 0 |
| 0.60 G 0.40 T | 0 | 0 | 2410 | 0 | 0 | 0 | 2410 | 0 |
| 0.65 G 0.35 T | 0 | 0 | 2410 | 0 | 0 | 0 | 2410 | 0 |
| 0.70 G 0.30 T | 0 | 0 | 2410 | 0 | 0 | 0 | 2410 | 0 |
| 0.75 G 0.25 T | 0 | 0 | 2410 | 0 | 0 | 0 | 2410 | 0 |
| 0.80 G 0.20 T | 0 | 0 | 2410 | 0 | 0 | 0 | 2410 | 0 |
| 0.85 G 0.15 T | 0 | 0 | 2410 | 0 | 0 | 0 | 2410 | 0 |
| 0.90 G 0.10 T | 0 | 0 | 2410 | 0 | 0 | 0 | 2410 | 0 |
| 0.95 G 0.05 T | 0 | 0 | 2410 | 0 | 0 | 0 | 2410 | 0 |

**Table A.9. Quantity purchased from each supplier in period 9 in case A and case B**

| Scenario | Case A | | | | Case B | | | |
| --- | --- | --- | --- | --- | --- | --- | --- | --- |
|  | S1 | S2 | S3 | S4 | S1 | S2 | S3 | S4 |
| 0.05 G 0.95 T | 0 | 0 | 0 | 515 | 0 | 0 | 0 | 515 |
| 0.1 G 0.9 T | 0 | 0 | 0 | 515 | 0 | 0 | 0 | 515 |
| 0.15 G 0.85 T | 0 | 0 | 0 | 515 | 0 | 0 | 0 | 515 |
| 0.2 G 0.8 T | 0 | 0 | 0 | 515 | 0 | 0 | 0 | 515 |
| 0.25 G 0.75 T | 0 | 0 | 0 | 515 | 0 | 0 | 0 | 515 |
| 0.3 G 0.7 T | 0 | 0 | 0 | 515 | 0 | 0 | 0 | 515 |
| 0.35 G 0.65 T | 0 | 0 | 0 | 515 | 0 | 0 | 0 | 515 |
| 0.40 G 0.60 T | 0 | 0 | 0 | 515 | 0 | 0 | 0 | 515 |
| 0.45 G 0.55 T | 0 | 0 | 0 | 515 | 0 | 0 | 0 | 515 |
| 0.50 G 0.5 T | 0 | 0 | 0 | 515 | 0 | 0 | 0 | 515 |
| 0.55 G 0.45 T | 0 | 0 | 0 | 515 | 0 | 0 | 0 | 515 |
| 0.60 G 0.40 T | 0 | 0 | 0 | 515 | 0 | 0 | 0 | 515 |
| 0.65 G 0.35 T | 0 | 0 | 0 | 515 | 0 | 0 | 0 | 515 |
| 0.70 G 0.30 T | 0 | 0 | 515 | 0 | 0 | 0 | 515 | 0 |
| 0.75 G 0.25 T | 0 | 0 | 515 | 0 | 0 | 0 | 515 | 0 |
| 0.80 G 0.20 T | 0 | 0 | 515 | 0 | 0 | 0 | 515 | 0 |
| 0.85 G 0.15 T | 0 | 0 | 515 | 0 | 0 | 0 | 515 | 0 |
| 0.90 G 0.10 T | 0 | 0 | 515 | 0 | 0 | 0 | 515 | 0 |
| 0.95 G 0.05 T | 0 | 0 | 515 | 0 | 0 | 0 | 515 | 0 |

**Table A.10. Quantity purchased from each supplier in period 10 in case A and case B**

| Scenario | Case A | | | | Case B | | | |
| --- | --- | --- | --- | --- | --- | --- | --- | --- |
|  | S1 | S2 | S3 | S4 | S1 | S2 | S3 | S4 |
| 0.05 G 0.95 T | 0 | 0 | 0 | 1850 | 0 | 0 | 0 | 1850 |
| 0.1 G 0.9 T | 0 | 0 | 0 | 1850 | 0 | 0 | 0 | 1850 |
| 0.15 G 0.85 T | 0 | 0 | 0 | 1850 | 0 | 0 | 0 | 1850 |
| 0.2 G 0.8 T | 0 | 0 | 0 | 1850 | 0 | 0 | 0 | 1850 |
| 0.25 G 0.75 T | 0 | 0 | 0 | 1850 | 0 | 0 | 0 | 1850 |
| 0.3 G 0.7 T | 0 | 0 | 0 | 1850 | 0 | 0 | 0 | 1850 |
| 0.35 G 0.65 T | 0 | 0 | 0 | 1850 | 0 | 0 | 0 | 1850 |
| 0.40 G 0.60 T | 0 | 0 | 0 | 1850 | 0 | 0 | 0 | 1850 |
| 0.45 G 0.55 T | 0 | 0 | 0 | 1850 | 0 | 0 | 0 | 1850 |
| 0.50 G 0.5 T | 0 | 0 | 0 | 1850 | 0 | 0 | 0 | 1850 |
| 0.55 G 0.45 T | 0 | 0 | 0 | 1850 | 0 | 0 | 0 | 1850 |
| 0.60 G 0.40 T | 0 | 0 | 0 | 1850 | 0 | 0 | 0 | 1850 |
| 0.65 G 0.35 T | 0 | 0 | 1850 | 0 | 0 | 0 | 1850 | 0 |
| 0.70 G 0.30 T | 0 | 0 | 1850 | 0 | 0 | 0 | 1850 | 0 |
| 0.75 G 0.25 T | 0 | 0 | 1850 | 0 | 0 | 0 | 1850 | 0 |
| 0.80 G 0.20 T | 0 | 0 | 1850 | 0 | 0 | 0 | 1850 | 0 |
| 0.85 G 0.15 T | 0 | 0 | 2170 | 0 | 0 | 0 | 1850 | 0 |
| 0.90 G 0.10 T | 0 | 0 | 2170 | 0 | 0 | 0 | 1850 | 0 |
| 0.95 G 0.05 T | 0 | 0 | 2170 | 0 | 0 | 0 | 1850 | 0 |

**Table A.11. Quantity purchased from each supplier in period 11 in case A and case B**

| Scenario | Case A | | | | Case B | | | |
| --- | --- | --- | --- | --- | --- | --- | --- | --- |
|  | S1 | S2 | S3 | S4 | S1 | S2 | S3 | S4 |
| 0.05 G 0.95 T | 0 | 0 | 0 | 320 | 0 | 0 | 0 | 320 |
| 0.1 G 0.9 T | 0 | 0 | 0 | 320 | 0 | 0 | 0 | 320 |
| 0.15 G 0.85 T | 0 | 0 | 0 | 320 | 0 | 0 | 0 | 320 |
| 0.2 G 0.8 T | 0 | 0 | 0 | 320 | 0 | 0 | 0 | 320 |
| 0.25 G 0.75 T | 0 | 0 | 0 | 320 | 0 | 0 | 0 | 320 |
| 0.3 G 0.7 T | 0 | 0 | 0 | 320 | 0 | 0 | 0 | 320 |
| 0.35 G 0.65 T | 0 | 0 | 0 | 320 | 0 | 0 | 0 | 320 |
| 0.40 G 0.60 T | 0 | 0 | 0 | 320 | 0 | 0 | 0 | 320 |
| 0.45 G 0.55 T | 0 | 0 | 0 | 320 | 0 | 0 | 0 | 320 |
| 0.50 G 0.5 T | 0 | 0 | 0 | 320 | 0 | 0 | 0 | 320 |
| 0.55 G 0.45 T | 0 | 0 | 0 | 320 | 0 | 0 | 0 | 320 |
| 0.60 G 0.40 T | 0 | 0 | 0 | 320 | 0 | 0 | 0 | 320 |
| 0.65 G 0.35 T | 0 | 0 | 0 | 320 | 0 | 0 | 0 | 320 |
| 0.70 G 0.30 T | 0 | 0 | 0 | 320 | 0 | 0 | 0 | 320 |
| 0.75 G 0.25 T | 0 | 0 | 0 | 320 | 0 | 0 | 0 | 320 |
| 0.80 G 0.20 T | 0 | 0 | 0 | 320 | 0 | 0 | 0 | 320 |
| 0.85 G 0.15 T | 0 | 0 | 0 | **0** | 0 | 0 | 0 | **320** |
| 0.90 G 0.10 T | **0** | 0 | 0 | 0 | **320** | 0 | 0 | 0 |
| 0.95 G 0.05 T | **0** | 0 | 0 | 0 | **320** | 0 | 0 | 0 |

**Table A.12. Quantity purchased from each supplier in period 12 in case A and case B**

| Scenario | Case A | | | | Case B | | | |
| --- | --- | --- | --- | --- | --- | --- | --- | --- |
|  | S1 | S2 | S3 | S4 | S1 | S2 | S3 | S4 |
| 0.05 G 0.95 T | 0 | 0 | 540 | 0 | 0 | 0 | 540 | 0 |
| 0.1 G 0.9 T | 0 | 0 | 540 | 0 | 0 | 0 | 540 | 0 |
| 0.15 G 0.85 T | 0 | 0 | 540 | 0 | 0 | 0 | 540 | 0 |
| 0.2 G 0.8 T | 0 | 0 | 540 | 0 | 0 | 0 | 540 | 0 |
| 0.25 G 0.75 T | 0 | 0 | 540 | 0 | 0 | 0 | 540 | 0 |
| 0.3 G 0.7 T | 0 | 0 | 540 | 0 | 0 | 0 | 540 | 0 |
| 0.35 G 0.65 T | 0 | 0 | 540 | 0 | 0 | 0 | 540 | 0 |
| 0.40 G 0.60 T | 0 | 0 | 540 | 0 | 0 | 0 | 540 | 0 |
| 0.45 G 0.55 T | 0 | 0 | 540 | 0 | 0 | 0 | 540 | 0 |
| 0.50 G 0.5 T | 0 | 0 | 540 | 0 | 0 | 0 | 540 | 0 |
| 0.55 G 0.45 T | 0 | 0 | 540 | 0 | 0 | 0 | 540 | 0 |
| 0.60 G 0.40 T | 0 | 0 | 540 | 0 | 0 | 0 | 540 | 0 |
| 0.65 G 0.35 T | 0 | 0 | 540 | 0 | 0 | 0 | 540 | 0 |
| 0.70 G 0.30 T | 0 | 0 | 540 | 0 | 0 | 0 | 540 | 0 |
| 0.75 G 0.25 T | 0 | 0 | 540 | 0 | 0 | 0 | 540 | 0 |
| 0.80 G 0.20 T | 0 | 0 | 540 | 0 | 0 | 0 | 540 | 0 |
| 0.85 G 0.15 T | 0 | 0 | 540 | 0 | 0 | 0 | 540 | 0 |
| 0.90 G 0.10 T | 0 | 0 | 540 | 0 | 0 | 0 | 540 | 0 |
| 0.95 G 0.05 T | 0 | 0 | 540 | 0 | 0 | 0 | 540 | 0 |

**Table A.13. Quantity purchased from each supplier in period 13 in case A and case B**

| Scenario | Case A | | | | Case B | | | |
| --- | --- | --- | --- | --- | --- | --- | --- | --- |
|  | S1 | S2 | S3 | S4 | S1 | S2 | S3 | S4 |
| 0.05 G 0.95 T | 0 | 0 | 0 | 590 | 0 | 0 | 0 | 590 |
| 0.1 G 0.9 T | 0 | 0 | 0 | 590 | 0 | 0 | 0 | 590 |
| 0.15 G 0.85 T | 0 | 0 | 0 | 590 | 0 | 0 | 0 | 590 |
| 0.2 G 0.8 T | 0 | 0 | 0 | 590 | 0 | 0 | 0 | 590 |
| 0.25 G 0.75 T | 0 | 0 | 0 | 590 | 0 | 0 | 0 | 590 |
| 0.3 G 0.7 T | 0 | 0 | 0 | 590 | 0 | 0 | 0 | 590 |
| 0.35 G 0.65 T | 0 | 0 | 0 | 590 | 0 | 0 | 0 | 590 |
| 0.40 G 0.60 T | 0 | 0 | 0 | 590 | 0 | 0 | 0 | 590 |
| 0.45 G 0.55 T | 0 | 0 | 0 | 590 | 0 | 0 | 0 | 590 |
| 0.50 G 0.5 T | 0 | 0 | 0 | 590 | 0 | 0 | 0 | 590 |
| 0.55 G 0.45 T | 0 | 0 | 0 | 590 | 0 | 0 | 0 | 590 |
| 0.60 G 0.40 T | 0 | 0 | 0 | 590 | 0 | 0 | 0 | 590 |
| 0.65 G 0.35 T | 0 | 0 | 0 | **840** | 0 | 0 | 0 | **590** |
| 0.70 G 0.30 T | 0 | 0 | 590 | 0 | 0 | 0 | 590 | 0 |
| 0.75 G 0.25 T | 0 | 0 | **840** | 0 | 0 | 0 | **590** | 0 |
| 0.80 G 0.20 T | 0 | 0 | **840** | 0 | 0 | 0 | **590** | 0 |
| 0.85 G 0.15 T | 0 | 0 | 590 | 0 | 0 | 0 | 590 | 0 |
| 0.90 G 0.10 T | 0 | 0 | 590 | 0 | 0 | 0 | 590 | 0 |
| 0.95 G 0.05 T | 0 | 0 | **840** | 0 | 0 | 0 | **590** | 0 |

**Table A.14. Quantity purchased from each supplier in period 14 in case A and case B**

| Scenario | Case A | | | | Case B | | | |
| --- | --- | --- | --- | --- | --- | --- | --- | --- |
|  | S1 | S2 | S3 | S4 | S1 | S2 | S3 | S4 |
| 0.05 G 0.95 T | 0 | 0 | 0 | 250 | 0 | 0 | 0 | 250 |
| 0.1 G 0.9 T | 0 | 0 | 0 | 250 | 0 | 0 | 0 | 250 |
| 0.15 G 0.85 T | 0 | 0 | 0 | 250 | 0 | 0 | 0 | 250 |
| 0.2 G 0.8 T | 0 | 0 | 0 | 250 | 0 | 0 | 0 | 250 |
| 0.25 G 0.75 T | 0 | 0 | 0 | 250 | 0 | 0 | 0 | 250 |
| 0.3 G 0.7 T | 0 | 0 | 0 | 250 | 0 | 0 | 0 | 250 |
| 0.35 G 0.65 T | 0 | 0 | 0 | 250 | 0 | 0 | 0 | 250 |
| 0.40 G 0.60 T | 0 | 0 | 0 | 250 | 0 | 0 | 0 | 250 |
| 0.45 G 0.55 T | 0 | 0 | 0 | 250 | 0 | 0 | 0 | 250 |
| 0.50 G 0.5 T | 0 | 0 | 0 | 250 | 0 | 0 | 0 | 250 |
| 0.55 G 0.45 T | 0 | 0 | 0 | 250 | 0 | 0 | 0 | 250 |
| 0.60 G 0.40 T | 0 | 0 | 0 | 250 | 0 | 0 | 0 | 250 |
| 0.65 G 0.35 T | 0 | 0 | **0** | **0** | 0 | 0 | **250** | **0** |
| 0.70 G 0.30 T | 0 | 0 | 250 | 0 | 0 | 0 | 250 | 0 |
| 0.75 G 0.25 T | 0 | 0 | **0** | 0 | 0 | 0 | **250** | 0 |
| 0.80 G 0.20 T | 0 | 0 | **0** | 0 | 0 | 0 | **250** | 0 |
| 0.85 G 0.15 T | 0 | 0 | 250 | 0 | 0 | 0 | 250 | 0 |
| 0.90 G 0.10 T | 0 | 0 | 250 | 0 | 0 | 0 | 250 | 0 |
| 0.95 G 0.05 T | 0 | 0 | **0** | 0 | 0 | 0 | 250 | 0 |

**Table A.15. Quantity purchased from each supplier in period 15 in case A and case B**

| Scenario | Case A | | | | Case B | | | |
| --- | --- | --- | --- | --- | --- | --- | --- | --- |
|  | S1 | S2 | S3 | S4 | S1 | S2 | S3 | S4 |
| 0.05 G 0.95 T | 0 | 0 | 0 | 780 | 0 | 0 | 0 | 780 |
| 0.1 G 0.9 T | 0 | 0 | 0 | 780 | 0 | 0 | 0 | 780 |
| 0.15 G 0.85 T | 0 | 0 | 0 | 780 | 0 | 0 | 0 | 780 |
| 0.2 G 0.8 T | 0 | 0 | 0 | 780 | 0 | 0 | 0 | 780 |
| 0.25 G 0.75 T | 0 | 0 | 0 | 780 | 0 | 0 | 0 | 780 |
| 0.3 G 0.7 T | 0 | 0 | 0 | 780 | 0 | 0 | 0 | 780 |
| 0.35 G 0.65 T | 0 | 0 | 0 | 780 | 0 | 0 | 0 | 780 |
| 0.40 G 0.60 T | 0 | 0 | 0 | 780 | 0 | 0 | 0 | 780 |
| 0.45 G 0.55 T | 0 | 0 | 0 | 780 | 0 | 0 | 0 | 780 |
| 0.50 G 0.5 T | 0 | 0 | 0 | 780 | 0 | 0 | 0 | 780 |
| 0.55 G 0.45 T | 0 | 0 | 0 | 780 | 0 | 0 | 0 | 780 |
| 0.60 G 0.40 T | 0 | 0 | 0 | 780 | 0 | 0 | 0 | 780 |
| 0.65 G 0.35 T | 0 | 0 | 0 | 780 | 0 | 0 | 0 | 780 |
| 0.70 G 0.30 T | 0 | 0 | 780 | 0 | 0 | 0 | 780 | 0 |
| 0.75 G 0.25 T | 0 | 0 | 780 | 0 | 0 | 0 | 780 | 0 |
| 0.80 G 0.20 T | 0 | 0 | 780 | 0 | 0 | 0 | 780 | 0 |
| 0.85 G 0.15 T | 0 | 0 | 780 | 0 | 0 | 0 | 780 | 0 |
| 0.90 G 0.10 T | 0 | 0 | 780 | 0 | 0 | 0 | 780 | 0 |
| 0.95 G 0.05 T | 0 | 0 | 780 | 0 | 0 | 0 | 780 | 0 |

**Table A.16. Quantity purchased from each supplier in period 16 in case A and case B**

| Scenario | Case A | | | | Case B | | | |
| --- | --- | --- | --- | --- | --- | --- | --- | --- |
|  | S1 | S2 | S3 | S4 | S1 | S2 | S3 | S4 |
| 0.05 G 0.95 T | 0 | 0 | 830 | 0 | 0 | 0 | 830 | 0 |
| 0.1 G 0.9 T | 0 | 0 | 830 | 0 | 0 | 0 | 830 | 0 |
| 0.15 G 0.85 T | 0 | 0 | 830 | 0 | 0 | 0 | 830 | 0 |
| 0.2 G 0.8 T | 0 | 0 | 830 | 0 | 0 | 0 | 830 | 0 |
| 0.25 G 0.75 T | 0 | 0 | 830 | 0 | 0 | 0 | 830 | 0 |
| 0.3 G 0.7 T | 0 | 0 | 830 | 0 | 0 | 0 | 830 | 0 |
| 0.35 G 0.65 T | 0 | 0 | 830 | 0 | 0 | 0 | 830 | 0 |
| 0.40 G 0.60 T | 0 | 0 | 830 | 0 | 0 | 0 | 830 | 0 |
| 0.45 G 0.55 T | 0 | 0 | 830 | 0 | 0 | 0 | 830 | 0 |
| 0.50 G 0.5 T | 0 | 0 | 830 | 0 | 0 | 0 | 830 | 0 |
| 0.55 G 0.45 T | 0 | 0 | 830 | 0 | 0 | 0 | 830 | 0 |
| 0.60 G 0.40 T | 0 | 0 | 830 | 0 | 0 | 0 | 830 | 0 |
| 0.65 G 0.35 T | 0 | 0 | 830 | 0 | 0 | 0 | 830 | 0 |
| 0.70 G 0.30 T | 0 | 0 | 830 | 0 | 0 | 0 | 830 | 0 |
| 0.75 G 0.25 T | 0 | 0 | 830 | 0 | 0 | 0 | 830 | 0 |
| 0.80 G 0.20 T | 0 | 0 | 830 | 0 | 0 | 0 | 830 | 0 |
| 0.85 G 0.15 T | 0 | 0 | 830 | 0 | 0 | 0 | 830 | 0 |
| 0.90 G 0.10 T | 0 | 0 | 830 | 0 | 0 | 0 | 830 | 0 |
| 0.95 G 0.05 T | 0 | 0 | 830 | 0 | 0 | 0 | 830 | 0 |

**Table A.17. Quantity purchased from each supplier in period 17 in case A and case B**

| Scenario | Case A | | | | Case B | | | |
| --- | --- | --- | --- | --- | --- | --- | --- | --- |
|  | S1 | S2 | S3 | S4 | S1 | S2 | S3 | S4 |
| 0.05 G 0.95 T | 950 | 0 | 0 | 0 | 950 | 0 | 0 | 0 |
| 0.1 G 0.9 T | 950 | 0 | 0 | 0 | 950 | 0 | 0 | 0 |
| 0.15 G 0.85 T | 950 | 0 | 0 | 0 | 950 | 0 | 0 | 0 |
| 0.2 G 0.8 T | 950 | 0 | 0 | 0 | 950 | 0 | 0 | 0 |
| 0.25 G 0.75 T | 950 | 0 | 0 | 0 | 950 | 0 | 0 | 0 |
| 0.3 G 0.7 T | 950 | 0 | 0 | 0 | 950 | 0 | 0 | 0 |
| 0.35 G 0.65 T | 950 | 0 | 0 | 0 | 950 | 0 | 0 | 0 |
| 0.40 G 0.60 T | 950 | 0 | 0 | 0 | 950 | 0 | 0 | 0 |
| 0.45 G 0.55 T | 950 | 0 | 0 | 0 | 950 | 0 | 0 | 0 |
| 0.50 G 0.5 T | 950 | 0 | 0 | 0 | 950 | 0 | 0 | 0 |
| 0.55 G 0.45 T | 950 | 0 | 0 | 0 | 950 | 0 | 0 | 0 |
| 0.60 G 0.40 T | 950 | 0 | 0 | 0 | 950 | 0 | 0 | 0 |
| 0.65 G 0.35 T | 950 | 0 | 0 | 0 | 950 | 0 | 0 | 0 |
| 0.70 G 0.30 T | 950 | 0 | 0 | 0 | 950 | 0 | 0 | 0 |
| 0.75 G 0.25 T | 950 | 0 | 0 | 0 | 950 | 0 | 0 | 0 |
| 0.80 G 0.20 T | 950 | 0 | 0 | 0 | 950 | 0 | 0 | 0 |
| 0.85 G 0.15 T | 950 | 0 | 0 | 0 | 950 | 0 | 0 | 0 |
| 0.90 G 0.10 T | 950 | 0 | 0 | 0 | 950 | 0 | 0 | 0 |
| 0.95 G 0.05 T | 950 | 0 | 0 | 0 | 950 | 0 | 0 | 0 |

**Table A.18. Quantity purchased from each supplier in period 18 in case A and case B**

| Scenario | Case A | | | | Case B | | | |
| --- | --- | --- | --- | --- | --- | --- | --- | --- |
|  | S1 | S2 | S3 | S4 | S1 | S2 | S3 | S4 |
| 0.05 G 0.95 T | 0 | 0 | 0 | 1060 | 0 | 0 | 0 | 1060 |
| 0.1 G 0.9 T | 0 | 0 | 0 | **1060** | 0 | 0 | 0 | **650** |
| 0.15 G 0.85 T | 0 | 0 | 0 | **1060** | 0 | 0 | 0 | **650** |
| 0.2 G 0.8 T | 0 | 0 | 0 | **1060** | 0 | 0 | 0 | **650** |
| 0.25 G 0.75 T | 0 | 0 | 0 | **1060** | 0 | 0 | 0 | **650** |
| 0.3 G 0.7 T | 0 | 0 | 0 | **1060** | 0 | 0 | 0 | **650** |
| 0.35 G 0.65 T | 0 | 0 | 0 | 650 | 0 | 0 | 0 | 650 |
| 0.40 G 0.60 T | 0 | 0 | 0 | 650 | 0 | 0 | 0 | 650 |
| 0.45 G 0.55 T | 0 | 0 | 0 | 650 | 0 | 0 | 0 | 650 |
| 0.50 G 0.5 T | 0 | 0 | 0 | 650 | 0 | 0 | 0 | 650 |
| 0.55 G 0.45 T | 0 | 0 | 0 | 650 | 0 | 0 | 0 | 650 |
| 0.60 G 0.40 T | 0 | 0 | 0 | 650 | 0 | 0 | 0 | 650 |
| 0.65 G 0.35 T | 0 | 0 | **0** | **650** | 0 | 0 | **650** | **0** |
| 0.70 G 0.30 T | 0 | 0 | 650 | 0 | 0 | 0 | 650 | 0 |
| 0.75 G 0.25 T | 0 | 0 | 650 | 0 | 0 | 0 | 650 | 0 |
| 0.80 G 0.20 T | 0 | 0 | 650 | 0 | 0 | 0 | 650 | 0 |
| 0.85 G 0.15 T | 0 | 0 | 650 | 0 | 0 | 0 | 650 | 0 |
| 0.90 G 0.10 T | 0 | 0 | 650 | 0 | 0 | 0 | 650 | 0 |
| 0.95 G 0.05 T | 0 | 0 | 650 | 0 | 0 | 0 | 650 | 0 |

**Table A.19. Quantity purchased from each supplier in period 19 in case A and case B**

| Scenario | Case A | | | | Case B | | | |
| --- | --- | --- | --- | --- | --- | --- | --- | --- |
|  | S1 | S2 | S3 | S4 | S1 | S2 | S3 | S4 |
| 0.05 G 0.95 T | 0 | 0 | 0 | 0 | 0 | 0 | 0 | 0 |
| 0.1 G 0.9 T | 0 | 0 | **0** | 0 | 0 | 0 | **605** | 0 |
| 0.15 G 0.85 T | 0 | 0 | **0** | 0 | 0 | 0 | **605** | 0 |
| 0.2 G 0.8 T | 0 | 0 | **0** | 0 | 0 | 0 | **605** | 0 |
| 0.25 G 0.75 T | 0 | 0 | **0** | 0 | 0 | 0 | **605** | 0 |
| 0.3 G 0.7 T | 0 | 0 | **0** | 0 | 0 | 0 | **605** | 0 |
| 0.35 G 0.65 T | 0 | 0 | 605 | 0 | 0 | 0 | 605 | 0 |
| 0.40 G 0.60 T | 0 | 0 | 605 | 0 | 0 | 0 | 605 | 0 |
| 0.45 G 0.55 T | 0 | 0 | 605 | 0 | 0 | 0 | 605 | 0 |
| 0.50 G 0.5 T | 0 | 0 | 605 | 0 | 0 | 0 | 605 | 0 |
| 0.55 G 0.45 T | 0 | 0 | 605 | 0 | 0 | 0 | 605 | 0 |
| 0.60 G 0.40 T | 0 | 0 | 605 | 0 | 0 | 0 | 605 | 0 |
| 0.65 G 0.35 T | 0 | 0 | 605 | 0 | 0 | 0 | 605 | 0 |
| 0.70 G 0.30 T | 0 | 0 | 605 | 0 | 0 | 0 | 605 | 0 |
| 0.75 G 0.25 T | 0 | 0 | 605 | 0 | 0 | 0 | 605 | 0 |
| 0.80 G 0.20 T | 0 | 0 | 605 | 0 | 0 | 0 | 605 | 0 |
| 0.85 G 0.15 T | 0 | 0 | 605 | 0 | 0 | 0 | 605 | 0 |
| 0.90 G 0.10 T | 0 | 0 | 605 | 0 | 0 | 0 | 605 | 0 |
| 0.95 G 0.05 T | 0 | 0 | 605 | 0 | 0 | 0 | 605 | 0 |

**Table A.20. Quantity purchased from each supplier in period 20 in case A and case B**

| Scenario | Case A | | | | Case B | | | |
| --- | --- | --- | --- | --- | --- | --- | --- | --- |
|  | S1 | S2 | S3 | S4 | S1 | S2 | S3 | S4 |
| 0.05 G 0.95 T | 0 | 0 | 0 | 195 | 0 | 0 | 0 | 195 |
| 0.1 G 0.9 T | 0 | 0 | 0 | **195** | 0 | 0 | 0 | 0 |
| 0.15 G 0.85 T | 0 | 0 | 0 | **195** | 0 | 0 | 0 | 0 |
| 0.2 G 0.8 T | 0 | 0 | 0 | **195** | 0 | 0 | 0 | 0 |
| 0.25 G 0.75 T | 0 | 0 | 0 | **195** | 0 | 0 | 0 | 0 |
| 0.3 G 0.7 T | 0 | 0 | 0 | **195** | 0 | 0 | 0 | 0 |
| 0.35 G 0.65 T | 0 | 0 | 0 | 0 | 0 | 0 | 0 | 0 |
| 0.40 G 0.60 T | 0 | 0 | 0 | 0 | 0 | 0 | 0 | 0 |
| 0.45 G 0.55 T | 0 | 0 | 0 | 0 | 0 | 0 | 0 | 0 |
| 0.50 G 0.5 T | 0 | 0 | 0 | 0 | 0 | 0 | 0 | 0 |
| 0.55 G 0.45 T | 0 | 0 | 0 | 0 | 0 | 0 | 0 | 0 |
| 0.60 G 0.40 T | 0 | 0 | 0 | 0 | 0 | 0 | 0 | 0 |
| 0.65 G 0.35 T | 0 | 0 | 0 | 0 | 0 | 0 | 0 | 0 |
| 0.70 G 0.30 T | 0 | 0 | 0 | 0 | 0 | 0 | 0 | 0 |
| 0.75 G 0.25 T | 0 | 0 | 0 | 0 | 0 | 0 | 0 | 0 |
| 0.80 G 0.20 T | 0 | 0 | 0 | 0 | 0 | 0 | 0 | 0 |
| 0.85 G 0.15 T | 0 | 0 | 0 | 0 | 0 | 0 | 0 | 0 |
| 0.90 G 0.10 T | 0 | 0 | 0 | 0 | 0 | 0 | 0 | 0 |
| 0.95 G 0.05 T | 0 | 0 | 0 | 0 | 0 | 0 | 0 | 0 |
