# Supplementary material for: Datasets for supplier selection and order allocation with green criteria, all-unit quantity discounts and varying number of suppliers
Source: Data Brief. 2017 Jun 9;13:444–52. doi: 10.1016/j.dib.2017.06.018 (PMC5485863; doi:10.1016/j.dib.2017.06.018)
Supplement: Supplementary file 4 — Supplementary material Quantitiy discount vs. No quantity discount data (Table B.1 - Table B.5) [file mmc4.zip › Data_In_Brief/3.Qd vs No QD/QD vs no QD Output.docx]

Table B.1: Total optimal quantities ordered from each supplier (case A)

|  | Scenario | S1 | S2 | S3 | S4 |
| --- | --- | --- | --- | --- | --- |
| 1 | 0.05 G 0.95 T | 1610 | 0 | 4970 | 9110 |
| 2 | 0.1 G 0.9 T | 1610 | 0 | 4970 | 9110 |
| 3 | 0.15 G 0.85 T | 1610 | 0 | 4970 | 9110 |
| 4 | 0.2 G 0.8 T | 1610 | 0 | 4970 | 9110 |
| 5 | 0.25 G 0.75 T | 1610 | 0 | 4970 | 9110 |
| 6 | 0.3 G 0.7 T | 1610 | 0 | 4970 | 9110 |
| 7 | 0.35 G 0.65 T | 1610 | 0 | 5575 | 8505 |
| 8 | 0.40 G 0.60 T | 1610 | 0 | 5575 | 8505 |
| 9 | 0.45 G 0.55 T | 1610 | 0 | 5575 | 8505 |
| 10 | 0.50 G 0.5 T | 1610 | 0 | 5575 | 8505 |
| 11 | 0.55 G 0.45 T | 1610 | 0 | 5575 | 8505 |
| 12 | 0.60 G 0.40 T | 1610 | 0 | 5575 | 8505 |
| 13 | 0.65 G 0.35 T | 1610 | 0 | 7425 | 6655 |
| 14 | 0.70 G 0.30 T | 1610 | 0 | 12440 | 1640 |
| 15 | 0.75 G 0.25 T | 1610 | 0 | 12440 | 1640 |
| 16 | 0.80 G 0.20 T | 1610 | 0 | 12440 | 1640 |
| 17 | 0.85 G 0.15 T | 2930 | 0 | 12760 | 0 |
| 18 | 0.90 G 0.10 T | 2930 | 0 | 12760 | 0 |
| 19 | 0.95 G 0.05 T | 3250 | 0 | 12440 | 0 |

Table B.2: Total optimal quantities ordered from each supplier (after adding S5)

|  | Scenario | S1 | S2 | S3 | S4 | S5 | % Q lost from S3 |
| --- | --- | --- | --- | --- | --- | --- | --- |
| 1 | 0.05 G 0.95 T | 1610 | 0 | 2410 | 9110 | 2560 | 51.51% |
| 2 | 0.1 G 0.9 T | 1610 | 0 | 2410 | 9110 | 2560 | 51.51% |
| 3 | 0.15 G 0.85 T | 1610 | 0 | 2410 | 9110 | 2560 | 51.51% |
| 4 | 0.2 G 0.8 T | 1610 | 0 | 2410 | 9110 | 2560 | 51.51% |
| 5 | 0.25 G 0.75 T | 1610 | 0 | 2410 | 9110 | 2560 | 51.51% |
| 6 | 0.3 G 0.7 T | 1610 | 0 | 2410 | 8505 | 3165 | 51.51% |
| 7 | 0.35 G 0.65 T | 1610 | 0 | 2410 | 8505 | 3165 | 56.77% |
| 8 | 0.40 G 0.60 T | 1610 | 0 | 2410 | 8505 | 3165 | 56.77% |
| 9 | 0.45 G 0.55 T | 1610 | 0 | 2410 | 8505 | 3165 | 56.77% |
| 10 | 0.50 G 0.5 T | 1610 | 0 | 2410 | 8505 | 3165 | 56.77% |
| 11 | 0.55 G 0.45 T | 1610 | 0 | 2410 | 8505 | 3165 | 56.77% |
| 12 | 0.60 G 0.40 T | 1610 | 0 | 2410 | 8505 | 3165 | 56.77% |
| 13 | 0.65 G 0.35 T | 1610 | 0 | 4260 | 3150 | 6670 | 42.63% |
| 14 | 0.70 G 0.30 T | 1610 | 0 | 4260 | 1640 | 8180 | 65.76% |
| 15 | 0.75 G 0.25 T | 1610 | 0 | 4260 | 1640 | 8180 | 65.76% |
| 16 | 0.80 G 0.20 T | 1610 | 0 | 4260 | 1640 | 8180 | 65.76% |
| 17 | 0.85 G 0.15 T | 2930 | 0 | 4580 | 0 | 8180 | 64.11% |
| 18 | 0.90 G 0.10 T | 2930 | 0 | 4580 | 0 | 8180 | 64.11% |
| 19 | 0.95 G 0.05 T | 3250 | 0 | 4260 | 0 | 8180 | 65.76% |

Table B.3: Total optimal quantities ordered from each supplier (after adding S6)

|  | Scenario | S1 | S2 | S3 | S4 | S5 | S6 | % Q lost from S1 |
| --- | --- | --- | --- | --- | --- | --- | --- | --- |
| 1 | 0.05 G 0.95 T | 0 | 0 | 2410 | 9110 | 2560 | 1610 | 100% |
| 2 | 0.1 G 0.9 T | 0 | 0 | 2410 | 9110 | 2560 | 1610 | 100% |
| 3 | 0.15 G 0.85 T | 0 | 0 | 2410 | 9110 | 2560 | 1610 | 100% |
| 4 | 0.2 G 0.8 T | 0 | 0 | 2410 | 9110 | 2560 | 1610 | 100% |
| 5 | 0.25 G 0.75 T | 0 | 0 | 2410 | 9110 | 2560 | 1610 | 100% |
| 6 | 0.3 G 0.7 T | 0 | 0 | 2410 | 8505 | 3165 | 1610 | 100% |
| 7 | 0.35 G 0.65 T | 0 | 0 | 2410 | 8505 | 3165 | 1610 | 100% |
| 8 | 0.40 G 0.60 T | 0 | 0 | 2410 | 8505 | 3165 | 1610 | 100% |
| 9 | 0.45 G 0.55 T | 0 | 0 | 2410 | 8505 | 3165 | 1610 | 100% |
| 10 | 0.50 G 0.5 T | 0 | 0 | 2410 | 8505 | 3165 | 1610 | 100% |
| 11 | 0.55 G 0.45 T | 0 | 0 | 2410 | 8505 | 3165 | 1610 | 100% |
| 12 | 0.60 G 0.40 T | 0 | 0 | 2410 | 8505 | 3165 | 1610 | 100% |
| 13 | 0.65 G 0.35 T | 0 | 0 | 4260 | 3150 | 6670 | 1610 | 100% |
| 14 | 0.70 G 0.30 T | 0 | 0 | 4260 | 1640 | 8180 | 1610 | 100% |
| 15 | 0.75 G 0.25 T | 0 | 0 | 4260 | 1640 | 8180 | 1610 | 100% |
| 16 | 0.80 G 0.20 T | 0 | 0 | 4260 | 1640 | 8180 | 1610 | 100% |
| 17 | 0.85 G 0.15 T | 810 | 0 | 4580 | 0 | 8180 | 2120 | 72% |
| 18 | 0.90 G 0.10 T | 810 | 0 | 4580 | 0 | 8180 | 2120 | 72% |
| 19 | 0.95 G 0.05 T | 1130 | 0 | 4260 | 0 | 8180 | 2120 | 65% |

Table B.4: Total optimal quantities ordered from each supplier (after adding S7)

|  | Scenario | S1 | S2 | S3 | S4 | S5 | S6 | S7 | % Q lost from S4 |
| --- | --- | --- | --- | --- | --- | --- | --- | --- | --- |
| 1 | 0.05 G 0.95 T | 0 | 0 | 2410 | 4420 | 2560 | 1610 | 4690 | 51% |
| 2 | 0.1 G 0.9 T | 0 | 0 | 2410 | 4420 | 2560 | 1610 | 4690 | 51% |
| 3 | 0.15 G 0.85 T | 0 | 0 | 2410 | 4420 | 2560 | 1610 | 4690 | 51% |
| 4 | 0.2 G 0.8 T | 0 | 0 | 2410 | 3360 | 2970 | 1610 | 5340 | 63% |
| 5 | 0.25 G 0.75 T | 0 | 0 | 2410 | 3360 | 2970 | 1610 | 5340 | 63% |
| 6 | 0.3 G 0.7 T | 0 | 0 | 2410 | 3360 | 2970 | 1610 | 5340 | 60% |
| 7 | 0.35 G 0.65 T | 0 | 0 | 2410 | 3360 | 3165 | 1610 | 5145 | 60% |
| 8 | 0.40 G 0.60 T | 0 | 0 | 2410 | 3360 | 3165 | 1610 | 5145 | 60% |
| 9 | 0.45 G 0.55 T | 0 | 0 | 2410 | 3360 | 3165 | 1610 | 5145 | 60% |
| 10 | 0.50 G 0.5 T | 0 | 0 | 2410 | 3360 | 3165 | 1610 | 5145 | 60% |
| 11 | 0.55 G 0.45 T | 0 | 0 | 2410 | 3360 | 3165 | 1610 | 5145 | 60% |
| 12 | 0.60 G 0.40 T | 0 | 0 | 2410 | 3360 | 3165 | 1610 | 5145 | 60% |
| 13 | 0.65 G 0.35 T | 0 | 0 | 4260 | 1510 | 3165 | 1610 | 5145 | 52% |
| 14 | 0.70 G 0.30 T | 0 | 0 | 4260 | 0 | 8180 | 1610 | 1640 | 100% |
| 15 | 0.75 G 0.25 T | 0 | 0 | 4260 | 0 | 8180 | 1610 | 1640 | 100% |
| 16 | 0.80 G 0.20 T | 0 | 0 | 4260 | 0 | 8180 | 1610 | 1640 | 100% |
| 17 | 0.85 G 0.15 T | 0 | 0 | 4260 | 0 | 8180 | 1610 | 1640 |  |
| 18 | 0.90 G 0.10 T | 810 | 0 | 4580 | 0 | 8180 | 2120 | 0 |  |
| 19 | 0.95 G 0.05 T | 1130 | 0 | 4260 | 0 | 8180 | 2120 | 0 |  |

Table B.5: Total optimal quantities ordered from each supplier (after adding S8)

|  | Scenario | S1 | S2 | S3 | S4 | S5 | S6 | S7 | S8 | % Q lost from S1 |
| --- | --- | --- | --- | --- | --- | --- | --- | --- | --- | --- |
| 1 | 0.05 G 0.95 T | 0 | 0 | 2410 | 4420 | 2560 | 0 | 4690 | 1610 | 100% |
| 2 | 0.1 G 0.9 T | 0 | 0 | 2410 | 4420 | 2560 | 0 | 4690 | 1610 | 100% |
| 3 | 0.15 G 0.85 T | 0 | 0 | 2410 | 4420 | 2560 | 0 | 4690 | 1610 | 100% |
| 4 | 0.2 G 0.8 T | 0 | 0 | 2410 | 3360 | 2970 | 0 | 5340 | 1610 | 100% |
| 5 | 0.25 G 0.75 T | 0 | 0 | 2410 | 3360 | 2970 | 0 | 5340 | 1610 | 100% |
| 6 | 0.3 G 0.7 T | 0 | 0 | 2410 | 3360 | 2970 | 0 | 5340 | 1610 | 100% |
| 7 | 0.35 G 0.65 T | 0 | 0 | 2410 | 3360 | 3165 | 0 | 5145 | 1610 | 100% |
| 8 | 0.40 G 0.60 T | 0 | 0 | 2410 | 3360 | 3165 | 0 | 5145 | 1610 | 100% |
| 9 | 0.45 G 0.55 T | 0 | 0 | 2410 | 3360 | 3165 | 0 | 5145 | 1610 | 100% |
| 10 | 0.50 G 0.5 T | 0 | 0 | 2410 | 3360 | 3165 | 0 | 5145 | 1610 | 100% |
| 11 | 0.55 G 0.45 T | 0 | 0 | 2410 | 3360 | 3165 | 0 | 5145 | 1610 | 100% |
| 12 | 0.60 G 0.40 T | 0 | 0 | 2410 | 3360 | 3165 | 0 | 5145 | 1610 | 100% |
| 13 | 0.65 G 0.35 T | 0 | 0 | 4260 | 1510 | 3165 | 0 | 5145 | 1610 | 100% |
| 14 | 0.70 G 0.30 T | 0 | 0 | 4260 | 0 | 8180 | 0 | 1640 | 1610 | 100% |
| 15 | 0.75 G 0.25 T | 0 | 0 | 4260 | 0 | 8180 | 0 | 1640 | 1610 | 100% |
| 16 | 0.80 G 0.20 T | 0 | 0 | 4260 | 0 | 8180 | 0 | 1640 | 1610 | 100% |
| 17 | 0.85 G 0.15 T | 0 | 0 | 4260 | 0 | 8180 | 0 | 0 | 3250 | 100% |
| 18 | 0.90 G 0.10 T | 0 | 0 | 4260 | 0 | 8180 | 0 | 0 | 3250 | 100% |
| 19 | 0.95 G 0.05 T | 0 | 0 | 4260 | 0 | 8180 | 0 | 0 | 3250 | 100% |
